# Multiplexed single‐cell mass cytometry reveals distinct inhibitory effects on intracellular phosphoproteins by midostaurin in combination with chemotherapy in AML cells

**DOI:** 10.1186/s40164-021-00201-w

**Published:** 2021-02-02

**Authors:** Emma Rörby, Jörgen Adolfsson, Erik Hultin, Thomas Gustafsson, Kourosh Lotfi, Jörg Cammenga, Jan-Ingvar Jönsson

**Affiliations:** 1 Experimental Hematology Unit, Department of Biomedical and Clinical Sciences, Linköping University, 58185 Linköping, Sweden; 2 Science for Life Laboratory, National Mass Cytometry Facility, Linköping University, Linköping, Sweden; 3 Department of Hematology, Linköping University Hospital, Linköping, Sweden; 4 Clinical Pharmacology, Division of Drug Research, Department of Biomedical and Clinical Sciences, Linköping University, Linköping, Sweden

**Keywords:** Acute myeloid leukemia, Intracellular therapy response, Midostaurin, Chemotherapy, FLT3, Signaling proteins, Proteomics, Mass cytometry

## Abstract

**Background:**

Fms-related tyrosine kinase 3 (FLT3) receptor serves as a prognostic marker and therapeutic target in acute myeloid leukemia (AML). Approximately one-third of AML patients carry mutation in FLT3, associated with unfavourable prognosis and high relapse rate. The multitargeted kinase inhibitor midostaurin (PKC412) in combination with standard chemotherapy (daunorubicin and cytarabine) was recently shown to increase overall survival of AML patients. For that reason, PKC412 has been approved for treatment of AML patients with FLT3-mutation. PKC412 synergizes with standard chemotherapy, but the mechanism involved is not fully understood and the risk of relapse is still highly problematic.

**Methods:**

By utilizing the unique nature of mass cytometry for single cell multiparameter analysis, we have explored the proteomic effect and intracellular signaling response in individual leukemic cells with internal tandem duplication of FLT3 (FLT3-ITD) after midostaurin treatment in combination with daunorubicin or cytarabine.

**Results:**

We have identified a synergistic inhibition of intracellular signaling proteins after PKC412 treatment in combination with daunorubicin. In contrast, cytarabine antagonized phosphorylation inhibition of PKC412. Moreover, we found elevated levels of FLT3 surface expression after cytarabine treatment. Interestingly, the surface localization of FLT3 receptor increased *in vivo* on the blast cell population of two AML patients during day 3 of induction therapy (daunorubicin; once/day from day 1–3 and cytarabine; twice/day from day 1–7). We found FLT3 receptor expression to correlate with intracellular cytarabine (AraC) response. AML cell line cultured with AraC with or without PKC412 had an antagonizing phosphorylation inhibition of pAKT (p = 0.042 and 0.0261, respectively) and pERK1/2 (0.0134 and 0.0096, respectively) in FLT3^high^ compared to FLT3^low^ expressing cell populations.

**Conclusions:**

Our study provides insights into how conventional chemotherapy affects protein phosphorylation of vital signaling proteins in human leukemia cells. The results presented here support further investigation of novel strategies to treat FLT3-mutated AML patients with PKC412 in combination with chemotherapy agents and the potential development of novel treatment strategies.

## Introduction

Acute myeloid leukemia (AML) is a heterogeneous disease and mutations in the gene encoding fms-related tyrosine kinase 3 (FLT3; CD135) is one of the most common negative prognostic markers in AML, present in 20 % of all pediatric AML patients and 30 % of all adult AML patients [1–4]. FLT3 tandem duplication (FLT3-ITD) is a mutation that results in constitutive activation of FLT3, which provides a survival advantage of leukemic cells by activation of multiple downstream effector molecules. FLT3-ITD is associated with high relapse rates, resistance to chemotherapy and poor overall survival of AML patients [5, 6]. Mutations in the FLT3 gene were first thought to be easy drug targets, which resulted in the development of multiple receptor tyrosine kinase inhibitors (TKIs). TKIs targeting FLT3 however, generally elicit only partial and transient responses coupled to detection of drug-resistant leukemic cells [3]. Clinical trials investigating the use of first-generation FLT3 inhibitors in patients with AML often tested them as single-agents alone and even though the numbers of leukemic blasts were reduced patients rarely showed complete remission [7, 8]. Midostaurin (PKC412), a multitargeted kinase inhibitor, was originally developed to treat patients with solid tumors. Interestingly, recent preclinical studies determined a synergy between PKC412 and standard chemotherapy (daunorubicin and cytarabine) resulting in increased overall survival in AML patients [9, 10]. These findings support the notion that inhibition of FLT3 is important and emphasize the clinical effectiveness of PKC412 as a therapeutic agent to treat patients with AML. The efficacy of PKC412 in combination with standard chemotherapy lead to that the US Food and Drug Administration (FDA) in 2017 approved PKC412 (Rydapt, Novartis Pharmaceuticals, Inc) for treatment of patients with newly diagnosed FLT3-mutated AML and is the first drug to receive regulatory approval for AML since year 2000 in the United States [11]. In September the same year the European Medicines Agency (EMA) also approved PKC412 for use in combination therapy [12].

PKC412 has been shown to achieve the maximum clinical benefit when administered in combination with other anticancer agents, but the mechanism of action is not fully understood.

In this study, we sought to explore the downstream pathways that could play a role in the synergistic effect of PKC412. We reasoned that proteomic analyses offer a substantial advantage identifying post-transcriptional mechanisms and changes in signaling pathways [13, 14]. In order to circumvent the heterogeneity of AML, we applied single-cell resolution by mass cytometry, which allows for quantitative, high-throughput analysis of multiple cell surface markers as well as intracellular markers in single cells. Our results show that PKC412 in combination with daunorubicin has a synergistic inhibitory effect on several phosphoproteins important for cell proliferation and survival. Contrary, cytarabine antagonized the inhibition of protein phosphorylation by PKC412 and was shown to elevate the cell surface expression of FLT3. Moreover, FLT3 was upregulated in vivo during first days of chemotherapy treatment of AML patients (7 + 3 cytarabine and daunorubicin cycle).

Our study provide insights into how conventional chemotherapy affects protein phosphorylation of major signaling proteins in human leukemia cells and could reveal mechanism important to improve their use of chemotherapy for treatment of FLT3 mutated leukemic cells.

## Methods

### Cell lines and cell culture

Two leukemia cell lines with FLT3-ITD were included in this study: MOLM-13, heterozygous for FLT3-ITD (DSMZ, Braunschweig, Germany) and MV4-11 with FLT3 loss of heterozygosity, monoclonal for FLT3-ITD (ATCC, Manassan, VA, USA). Cell lines were maintained as exponentially growing cultures in 75 cm^2^ flasks, sub-cultured twice weekly. MOLM-13 cells were cultured in Roswell Park Memorial Institute (RPMI 1640; A1049101, Gibco, ThermoFisher Scientific, Gothenburg, Sweden) with 10 % fetal calf serum (FCS; Hyclone, ThermoFisher Scientific) and 1 % penicillin-streptomycin (Lonza, Verviers, Belgium). MV4-11 cells were cultured in Iscove´s modified Dulbecco's medium (IMDM, Lonza) with 10 % FCS (Hyclone, ThermoFisher Scientific), 2 nM l-glutamine (PPA Laboratories, Pashing, Austria) and 1 % penicillin-streptomycin (Lonza). Cell lines were incubated at 37 °C in 5 % CO2 and tested for mycoplasma contamination with a qPCR-based service (Eurofins Genomics, Ebersberg, Germany) on a regular basis.

### Patient material

The selected AML patients were confirmed FLT3-ITD positive in diagnostic routine, and ITD was detected by fragment analysis at allelic ratio > 0.7. Blood samples at time of diagnosis (day 0) and at early morning at day 3 during induction therapy (daunorubicin and cytarabine) was collected for mass cytometry analysis. Blood from 2 healthy donors was collected as control. To ensure the consistency and accuracy of mass cytometric analysis, samples were collected immediately after blood aspiration (< 1 min) into Smart Tube Proteomic Stabilizer kit (Smart Tube Inc, San Carlos, CA, USA). 1 mL of blood was mixed with 1.4 mL of Smart Tube Proteomic Stabilizer 1, incubated for 10 min at room temperature and then frozen at − 80 °C until the time of analysis. The study was approved by the Medical Research Ethical Committee and the institutional review board of the Linköping University Hospital. Participants gave written informed consent.

Mononuclear cells from one de novo AML blood sample (confirmed FLT3-ITD and NPM1 positive in diagnostic routine) for in vitro experiments were enriched by density centrifugation and cryopreserved. After thawing, cells were incubated in IMDM (Lonza) supplemented with 20 % FCS (Hyclone) for 24 h in 37 °C followed by 18 h in vitro treatment as indicated (untreated control, PKC412 50 nM, Daunorubicin 0.25 µM, Cytarabine 25 µM alone or in combinations). Cells were collected for CyTOF-analysis in Smart Tube Proteomic Stabilizer kit (Smart Tube Inc.) 1 mL of cell was mixed with 1.4 mL of Smart Tube Proteomic Stabilizer 1 and incubated for 10 min in room temperature and then frozen at − 80 °C.

### Chemical compounds

We cultured cells with or without PKC412 (Midostaurin; Sigma Aldrich, St Louis, MO, USA) alone or together (co-incubation) with daunorubicin (Scandinavian Medical Service, Helsingborg, Sweden) or cytarabine (Sigma). Cells were also cultured in daunorubicin alone and cytarabine alone. Stock solutions of 10 nM were prepared in water for all drugs and stored at − 20 °C, aliquoted to avoid repeated freeze-thawing. Final concentrations of compounds and time of incubation are indicated in the figures and/or figure text.

### Proteomic analysis by mass cytometry

Samples were thawed in a 10 °C water bath and washed with 7.6 mL of 1x Thaw-Lyse Buffer (Smart Tube Inc), centrifuged (600G, 5 min, 20 °C) and the pellet resuspended in PBS (Fluidigm, San Francisco, CA) containing 2 % FCS (Hyclone, ThermoFisher Scientific). Sample barcoding was accomplished using the Fluidigm Cell-ID 20-Plex Pd barcoding kit (Fluidigm). Individual samples were incubated with MaxPar Fix I (Fluidigm) for 10 min at room temperature, washed and resuspended in barcode perm buffer (Fluidigm), barcodes were then added to each sample and the solution incubated for 30 min at room temperature. Blocking of unspecific binding was accomplished through sample incubation with Mouse IgG1 (1:10) (BioLegend, San Diego, CA, USA) and Human TruStain FcX™ (Fc Receptor Blocking Solution, 1:10) (BioLegend) for 5 min at room temperature before staining with surface antibodies (Additional file 1: Table S1A) for 30 min at 4 °C. Intracellular staining was performed by incubation of samples in PBS (Fluidigm) containing 1 % paraformaldehyde (PFA; ThermoFisher Scientific) for 20 min in room temperature, followed by cell permeabilization in ice cold methanol (Sigma) for 10 min and intracellular blocking with Rabbit IgG1 (1:10) (Invitrogen) and Mouse IgG1 (1:10) (BioLegend) for 2 min at room temperature. We then stained intracellular components (Additional file 1: Table S2B) for 60 min at room temperature. Following staining samples were resuspended in PBS (Fluidigm) containing 4 % PFA (ThermoFisher Scientific), stored at 4 °C overnight in the dark, washed and resuspended in CSB (Fluidigm) containing 2 % Iridium for 30 min in room temperature before being washed with ddH2O. Samples were then resuspended in ddH2O containing 3.3 × 10^4^ EQ four element calibration beads/ 10^6^ cells (Fluidigm) and volume adjusted to 5 × 10^5^ cells/mL. Cells were then filtered and analyzed in a CyTOF2 (Fluidigm) instrument using a flow speed of 0.045 mL/min, a 30-s acquisition delay, and a 10-s detector stability delay. The Mass cytometry work was performed at the Science for Life Laboratory node at Linköping’s University, Linköping, Sweden.

### Flow cytometry

For flow cytometry analysis, blocking of unspecific binding was first accomplished through sample incubation with Human TruStain FcX™ (Fc Receptor Blocking Solution, 1:20) (BioLegend) followed by incubation with allophyocyanin (APC)-conjugated hCD135 (1:100, BioLegend; clone BV10A4H2). Mouse IgG_1_-APC was used as isotype control and dead cells were detected by staining (1:4000) with Fixable Viability Stain 520 (FVS-FITC, BD Bioscience). Cells were analyzed using FACSCanto™ II (BD Biosciences). Resulting data was re-analyzed using FlowJo software (FlowJo LLC, Ashland, OR, USA). For imaging flow cytometry, we used ImageStreamX MkII system (AMNIS, Seattle, USA) with Inspire software for data acquisition (AMNIS, Seattle, USA). Data analysis was performed with IDEAS software (AMNIS, Seattle, USA), after color compensation cells in aspect ratio 0.93–0.97 were further analyzed.

For inhibition state analysis in MV4-11 cells according to the manufacturer´s protocol (BD Bioscience), 18 h cultured cells were fixed by adding 1 mL of pre-warmed (37 °C) Fix/Lys-buffer (BD Bioscience) to the cell suspension. Cells were then permeabilized with Perm buffer III (558,050, BD Bioscience) followed by staining with Alexa Fluor 647 anti-ERK1/2 (pY202/pY204, BD Bioscience), we used BD Phospho-flow Alexa Fluor 647 anti-mouse IgG_1_ (BD Bioscience) as isotype control. Cells were analyzed using FACSCanto™ II (BD Biosciences). Resulting data was re-analyzed using FlowJo software (FlowJo LLC, Ashland, OR, USA).

### Apoptotic studies

Annexin V was used for the quantitation of apoptotic cells. MV4-11 or MOLM-13 cells were cultured for 18–48 h (n = 3). Cells were washed in cold phosphate-buffered saline (PBS, Invitrogen, Carlsbad, CA) and resuspended in staining buffer containing Alexa Flour 647-conjugated AnnexinV (Nordic BioSite, Täby, Sweden) according to the manufacturer´s protocol (BD Bioscience). Cells were incubated for 15 min and analyzed for AnnexinV-binding by flow cytometry on FACSCanto™ II (BD Biosciences).

### Statistical analysis

Data were analyzed using paired Students t test or Mann-Whitney test in Prism 7 (GraphPad Software). *P* < 0.05 was considered significant.

## Results

### Daunorubicin and cytarabine treatment have opposing effects on the phosphorylation status of signaling proteins when combined with PKC412

**Fig. 1:**
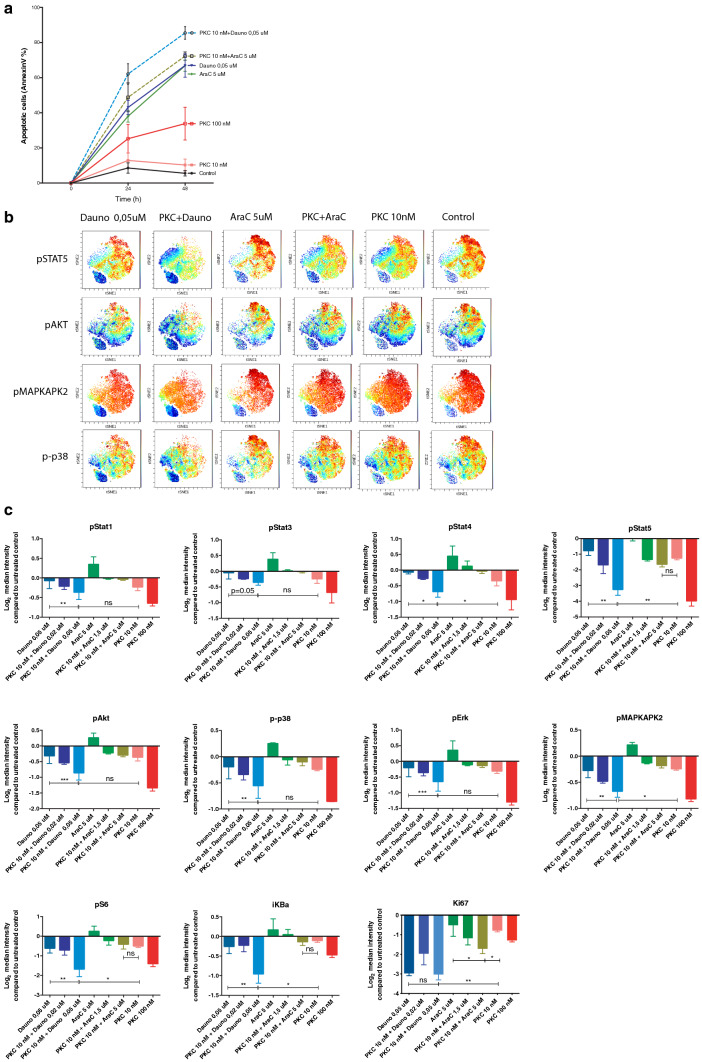
Mass cytometry defines synergistic phosphoprotein inhibition in co-cultures of midostaurin, daunorubicin and cytarabine. **a** Daunorubicin (Dauno) and cytarabine (AraC) in co-culture with midostaurin (PKC412; PKC). Average percentage of apoptotic MV4-11 cells (AnnexinV+) determined after 24 and 48 h of culture with midostaurin 10 and 100 nM (PKC412; pink and red line, respectively), daunorubicin 0.05 µM (Dauno; dark blue line) or co-culture with PKC412 (10 nM) plus Dauno (0.05 µM, PKC412 + Dauno; light blue dotted line), cytarabine 5 µM (AraC; dark green line) or PKC412 10 nM plus AraC 5 µM (PKC412 + AraC; light green dotted line). Untreated cells were used as control (black line). **b** viSNE was used to analyze MV4-11 cells cultured in vitro for 18 h with indicated inhibitors or untreated control. viSNE analysis was run on 6000 live intact single cells per sample. viSNE maps define cell populations based on intracellular markers, red indicate high expression and blue low expression. **c** Graph shows quantified change in expression illustrated as log_2_ median intensity compared to untreated control. PKC412 10 and 100 nM (pink and red bar), Dauno 0.05 µM (dark blue bar) or co-culture with PKC412 10 nM plus Dauno 0.05 µM (PKC412 + Dauno; light blue bar), AraC 5 µM (dark green bar) or PKC412 10 nM plus AraC 5 µM (PKC412 + AraC; light green bar). Mean ± SD (n = 3). *, *P* ≤ 0.05 **, *P* ≤ 0.01, as measured by paired Student’s *t* test

Both pre-clinical as well as clinical data demonstrate synergistic effects of PKC412 and standard chemotherapy [9, 10, 15, 16]. To verify the apoptotic effect of PKC412 in co-treatment with daunorubicin in FLT3-ITD expressing MV4-11 cells, we measured Annexin V binding after 24 and 48 h of culture (Fig. 1a). After 24 h of culture, 12.9 % of the PKC412-treated cells (10 nM) and 43.0 % of the daunorubicin-treated cells (0.05 µM) were apoptotic, while cells co-treated with both drugs were significantly more apoptotic and increased to 62.1 % (Fig. 1a). Since standard induction therapy also is composed of cytarabine [17], we also determined how co-incubation of PKC412 with cytarabine affected apoptosis. The percentage of apoptotic cells after 24 and 48 h of culture increased. As single drugs, cytarabine (5 µM) gave 38.0 % and PKC412 (10 nM) 12.9 % Annexin-V positive cells, while the combination resulted in an adaptive increase to 48.8 % apoptotic cells (Fig. 1a).

As PKC412 is a multitarget kinase inhibitor [18], we next decided to investigate how co-treatment with daunorubicin and cytarabine affected the phosphorylation of major signaling proteins. Constitutive activation of signaling proteins is frequently demonstrated in AML, comprising major anti-apoptotic as well as growth-regulating signaling cascades such as the RAF/MEK/ERK (Mitogen-activated protein kinase; MAPK) pathway, the phosphatidylinositol 3-kinase (PI3K)/AKT/mTOR pathway, and the JAK/STAT pathway [3, 19–21]. Since signals delivered by cytokines or by mutated receptors (e.g. FLT3) converge on proteins belonging to the these signaling pathways, we determined the phosphorylation status of several representative key molecules (e.g., STAT1, STAT3, STAT4, STAT5, AKT, ERK 1/2, IκBα, MAPKAPK2, p38 and S6) in MV4-11 by mass cytometry upon treatment with chemotherapeutic drugs. Ki67, a marker for different stages of active phases of the cell cycle, was also included as a proliferation marker. Eighteen hours of co-incubation with daunorubicin and PKC412 led to synergistically decreased phosphorylation of several proteins compared to untreated control cells (Fig. 1b, c). A viSNE map at single-cell resolution [22] shows a distinct downregulation of phosphorylated proteins in single PKC412-treated and daunorubicin-treated cells, which was even more profound after co-incubation with both drugs (Fig. 1b). Quantification of the data illustrates a significant decrease of phosphorylated STAT5 and MAPKAPK2 as well as STAT4, S6, and iKBa (Fig. 1c). The combination did not synergistically decrease the expression of Ki67, indicating that the cell cycle was not further affected by co-treatment with PKC412. When titrating the drugs, using gradually lower concentrations of daunorubicin and cytarabine, a stepwise effect on the expression of phosphorylated proteins could be demonstrated for both drugs co-incubated with PKC412 (Fig. 1c). Shorter incubation time (2 h) did not lead to any significant effect after co-incubation of PKC412 and daunorubicin (data not shown).

In contrast to the results with daunorubicin, the addition of cytarabine resulted in an increased phosphorylation of multiple signaling proteins compared to untreated control except for STAT5 after 18 h of culture (Fig. 1b, c). When cytarabine was added together with PKC412, it resulted partially in an antagonistic inhibitory effect on the phosphorylation of several signaling proteins including STAT1, STAT4, MAPKAPK2, p38, and ERK (Fig. 1b, c). However, the expression of Ki67 adaptively decreased when PKC412 was added together with cytarabine. Taken together, these results demonstrate that PKC412 in combination with daunorubicin has synergistic/additive inhibitory effects on the phosphorylation of major cell signaling proteins, whereas cytarabine counteracts the inhibitory effect of PKC412.

### Cytarabine increases the membrane localization of FLT3 of MV4-11 cells

**Fig. 2:**
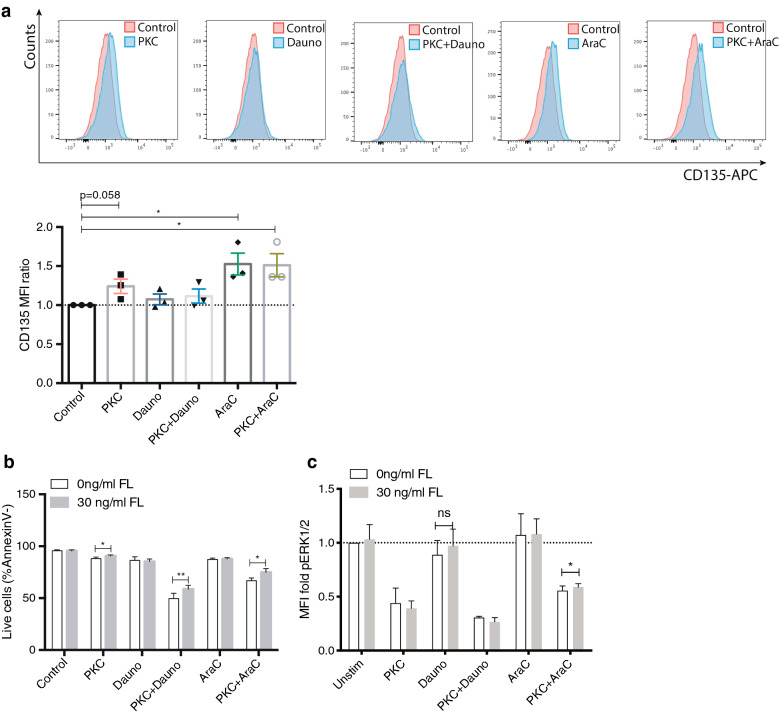
Cytarabine increase surface localization of FLT3 of MV4-11 cells. **a** Representative flow cytometry plots and bar graph showing the FLT3/CD135 surface expression of on MV4-11 cells after 18 h of culture with indicated treatment; PKC412 10 nM, Dauno 0.05 µM, PKC412 (10 nM) plus Dauno (0.05 µM), AraC 5 µM, PKC412 (10 nM) plus AraC 5 (µM), n = 3. **b** Percentage live cells (AnnexinV-) with or without FLT3 ligand (FL) after 18 h treatment, treatment as indicated; PKC412 50 nM, Dauno 0.05 µM, PKC412 (50 nM) plus Dauno (0.05 µM), AraC 5 µM, PKC412 (50 nM) plus AraC 5 µM, (n = 4) **c** Phospho-flow analysis of pERK1/2 expression 18 h after treatment start. Graph shown MFI fold expression compared to control (0 ng/mL FL). PKC412 50 nM, Dauno 0.05 µM, PKC412 50 nM plus Dauno 0.05 µM, AraC 5 µM, PKC412 50 nM plus AraC 5 µM. Mean ± SD (n = 3). *, *P* ≤ 0.05, as measured by Student’s *t* test

Since TKI AC220 (quizartinib) and PKC412 have both been shown to increase the cell surface localization of FLT3 [23, 24], we next determined whether treatment with PKC412 in combination with either daunorubicin or cytarabine affected FLT3 expression. FLT3-ITD positive MV4-11 cells were treated with PKC412 with or without daunorubicin or cytarabine, respectively, and analyzed by flow cytometry for FLT3 expression. Interestingly, the expression of FLT3 was significantly increased after 24 h in cytarabine-treated cultures both as single agent and in combination with PKC412 (Fig. 2a). We could also confirm elevated expression levels of FLT3 after PKC412 (10 nM) treatment alone although lower concentrations of PKC412 compared to a previous report from Reiter et al. were used [23]. In contrast, daunorubicin did not significantly altered FLT3 expression of MV4-11 after 24 h of culture.

The FLT3 protein at the cell surface is activated upon binding of FLT3 ligand (FL). Given the fact that plasma levels of FL has been shown to rise after chemotherapy treatment in AML patients [25], and our results indicate that FLT3 is upregulated in cytarabine-treated cells (Fig. 2a), we next studied the effect of FL in MV4-11 cultures after treatment with drugs. Using 30 ng/mL of FL, we found 18-h of FL treatment to increase the number of viable cells in co-treated cultures (Fig. 2b). Applying phosphoflow analysis, we observed a significant increase in pERK1/2 expression 24 h after adding FL to the cytarabine plus PKC412 cultures. Interestingly, cytarabine treated cells with no addition of FL showed a trend of increased pERK1/2 expression similar to adding 30 ng/mL FL to control cells. The analysis confirms the results of mass cytometry, demonstrating synergistic phosphorylation effect of daunorubicin and PKC412, but an antagonistic effect of cytarabine and PKC412 (Fig. 2b, c).

### Daunorubicin and PKC412 synergistically inhibit phosphorylation in MOLM-13 cells

**Fig. 3:**
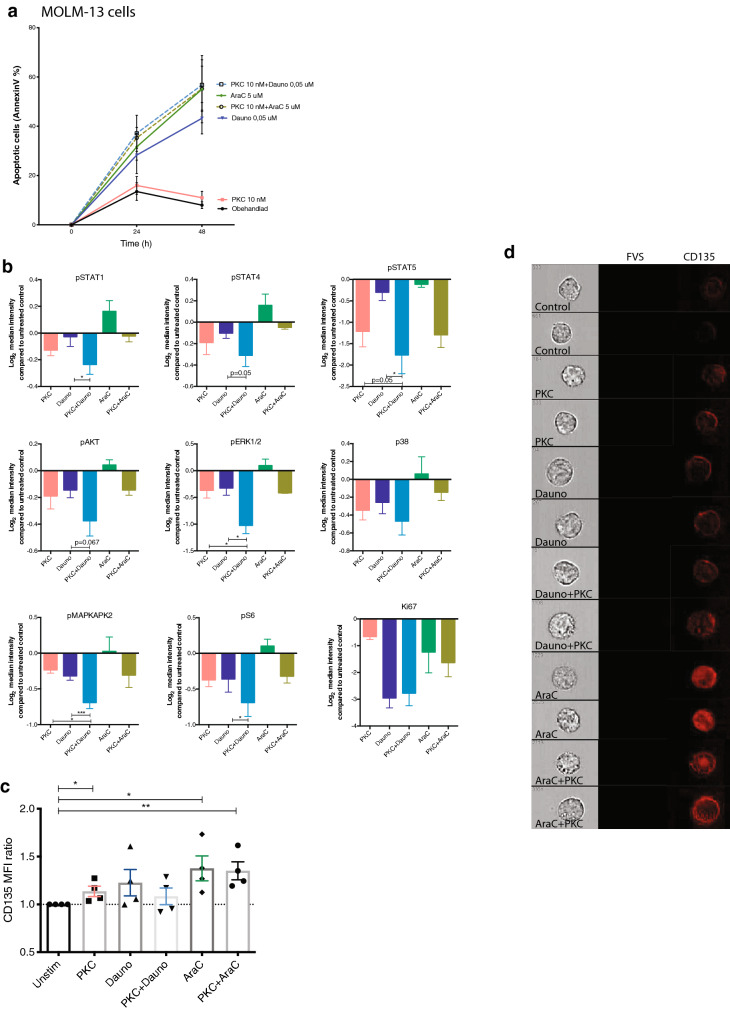
MOLM-13 cells exhibit synergistic phospho-inhibition upon co-incubation of PKC412 and daunorubicin. **a** Levels of apoptosis (AnnexinV+) in MOLM-13 cells 24 and 48 h of culture (n = 3). **b** Mass cytometry analysis of MOLM-13, quantified change in expression illustrated as log_2_ median intensity compared to control, n = 4. **c** Bar graph showing the FLT3/CD135 surface expression on MOLM-13 cells after 18 h of culture. Graph shown MFI fold expression compared to untreated control, n = 4. Treatment as indicated; PKC412 10 nM, Dauno 0.05 µM, PKC412 10 nM) plus Dauno 0.05 µM, AraC 5 µM, PKC412 10 nM plus AraC 5 µM. **d** ImageStream high resolution flow cytometry of FLT3/CD135. MOLM-13 cells were treated with PKC, daunorubicin (Dauno), cytarabine (AraC) or co-incubated as indicated in the figure. Fixable Viability Stain (FVS) were used to exclude dead cells. Mean ± SD, *, *P* ≤ 0.05 **, *P* ≤ 0.01, as measured by Student’s *t* test

**Fig. 4:**
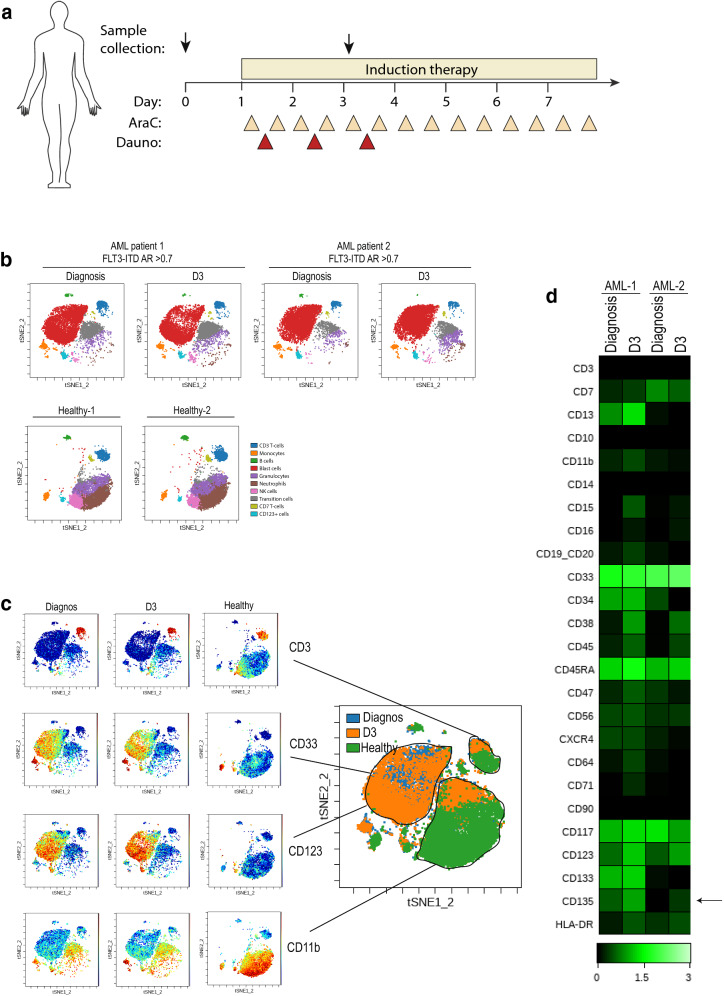
Induction chemotherapy increases FLT3 expression of AML patient cells in vivo. **a** Schematic of sampling strategy shown in (**b**). viSNE plots from time of diagnosis and day 3 of induction therapy (Dauno + AraC, 3 + 7) of AML patient-1, AML patient-2 both having FLT3-ITD allelic ratio (AR) > 0.7 and 2 healthy controls. viSNE maps manually gated cell populations based on 26 surface markers, populations as indicated in the map (blue; CD3 T-cells, orange; CD64 monocytes, green; CD19/CD20 B-cells, red; blast cells, purple; CD16 granulocytes, brown; CD15/CD16 neutrophils, pink; CD56 NK cells, grey; transition cells, yellow; CD7 T-cells, turquoise; CD123 + cells). viSNE analysis was run on 10,000 live intact single cells per sample. **c** Overlay of population for sample AML patient-1 at time of diagnosis and day 3 as well as healthy control overlaid to one viSNE map. **d** Heatmap of in vivo surface expression change during induction therapy in blast cell population, calculated transformed ratio of medians by row´s minimum using channel values (green correspond to high expression and black low). Arrow indicate FLT3/CD135 expression

Since the results above was collected from one cell line, we decided to evaluate the effect on phosphorylation after combined treatment in an additional FLT3-ITD positive cell line; MOLM-13. We could confirm the apoptotic effect after 24 and 48 h of culture (Fig. 3a) and PKC412 together with daunorubicin had an adaptive effect of apoptosis and led to either a synergistic or adaptive effect of phosphorylation of pSTAT5, pERK1/2 and pMAPKAPK2 (Fig. 4b). In contrast, PKC412 in combination with cytarabine could not increase apoptotic levels of MOLM-13 cells. However, similar to the result of MV4-11 cells, cytarabine antagonized the intracellular inhibitory function after 18 h of culture with PKC412, leading to increased expression of most phosphoproteins included in the analysis when compared to control, with the exception of pSTAT5 (Fig. 3b). Furthermore, cytarabine was shown to significantly enhance expression of FLT3, both as single agent and when co-incubated with PKC412 (Fig. 3c). Moreover, we confirmed the increased surface localization of FLT3 after cytarabine treatment using imagine flow cytometry (Fig. 3d).

Together these results demonstrate that the combination of PKC412 and daunorubicin inhibits phosphorylation of several phosphoproteins implied in FLT3-ITD signaling in either an adaptive or synergistic manner. In contrast, cytarabine antagonizes the intracellular inhibitory phosphorylation function of PKC412 and increases the surface localization of FLT3.

### Induction therapy increases FLT3 expression on leukemic blasts in AML patients already after 3 days of treatment

Given that we found cytarabine to upregulate FLT3 on leukemic cells in vitro, we decided to examine the FLT3 expression on leukemic blasts in AML patients undergoing induction therapy. Blood samples at time of diagnosis (day 0) and on day 3 were collected from two AML patients. Both patients had a FLT3-ITD allelic ratio (AR) > 0.7, and received first week of treatment with daunorubicin (once/day from day 1–3) and cytarabine (twice/day from day 1–7) as indicated from the experimental set up in Fig. 4a. To characterize the surface marker expression, we used the high dimensional resolution of mass cytometry to identify any differences between immunophenotypic distinct subpopulations. The analyses were performed based on 26 surface markers (listed in Additional file 1: Table S1A) including expression of CD3, CD33, CD123 and CD11b as seen in Fig. 4c. viSNE analysis revealed a massive population of blast cells in both AML patients compared to healthy individual as well as substantial immunophenotypic differences in AML blasts (Fig. 4b). The change in immunophenotypically-distinct populations is illustrated as an overlay when mapping patient sample from diagnosis and at day 3 as to healthy control in the same viSNE plot (Fig. 4c). Interestingly, FLT3 expression was shown to be elevated in AML blast cells during therapy (Fig. 4d, indicated by arrow). In the blast cell population, we found AML patient-1 to have a 2-fold increase in intensity of FLT3 expression during therapy and AML patient-2 to have a 1.6-fold increase (AML patient-1 at diagnosis having calculated raw value of FLT3 median: 2.78 and at D3 increased to: 5.57; AML patient-2 at diagnosis: 0 and D3: 1.63).

Taken together, the analyses confirm the results from cell lines in vitro, and demonstrate the effects of induction therapy on hematopoietic subpopulations *in vivo* of AML patients and indicate that FLT3 expression is elevated by intensive chemotherapy.

### FLT3 expression determines therapy response of cytarabine treated leukemic cells

**Fig. 5:**
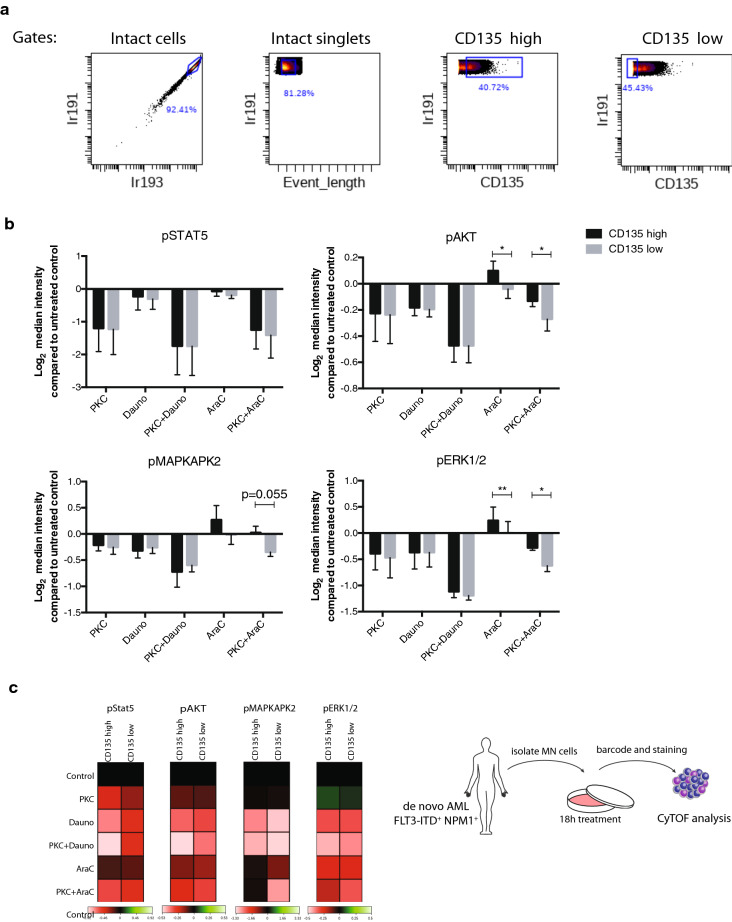
Surface expression of FLT3 determines therapy response. **a** Gating strategy of mass cytometry analysis for FLT3 high and FLT3 low expressing MOLM-13 cells after 18 h in vitro culture. **b** Graph showing quantified change in expression illustrated as log_2_ median intensity compared to control in FLT3 high and FLT3 low expressing MOLM-13 cells cultured with indicated treatments; PKC412 10 nM, Dauno 0.05 µM, PKC412 10 nM plus Dauno 0.05 µM, AraC 5 µM, PKC412 10 nM plus AraC 5 µM. ±SD (n = 4), *, *P* ≤ 0.05 **, *P* ≤ 0.01, as measured by Student’s *t* test. **c** Experimental set up and heatmap of in vitro intracellular change in phosphoprotein expression intensity during 18 h treatment (as indicated in the figure) in FLT3 high vs. FLT3 low expressing FLT3-ITD positive AML patient cells. Calculated transformed ratio of medians by first row using channel values (green correspond to high expression and red low)

To determine the correlation of FLT3 expression level to the response of chemotherapeutic drugs during treatment, we gated MOLM-13 cells treated in vitro into FLT3 high and low expressing populations, respectively (Fig. 5a). The levels of phosphoproteins involved in signaling were then determined separately in the two populations. Mass cytometry analysis revealed that the response to cytarabine treatment differed between FLT3 high and low expressing populations. Thus, an increase in phosphorylation during cytarabine treatment was clearly visible in the FLT3 high expressing cells, and the antagonistic inhibitory effect on phosphoproteins in combination with PKC412 was only seen in the FLT3 high expressing cells (Fig. 5b). Interestingly, in cultures with cytarabine with or without PKC412, expression of pSTAT5 was unchanged while other signaling proteins including pAKT and pERK1/2 showed significant higher expression in FLT3 high versus the low expressing cell population. In contrast, in PKC412 and daunorubicin treated cultures, the inhibitory effect was similar in both populations independent on FLT3 expression (Fig. 5b).

We also evaluated the effect of cytarabine in FLT3 high versus low expressing primary AML patient cells (FLT3-ITD positive, AR > 0.7) grown in vitro. Similarly to MOLM-13 cell line, based on FLT3 expression pERK1/2 and pMAPKAPK2 showed differences in phosphorylation status in PKC and cytarabine co-treated cultures while pSTAT5 was inhibited in similar manner in both FLT3 high and FLT3 low populations (Fig. 5c and Additional file 1: Table S2).

Conclusively, the effect of cytarabine leading to increase phosphorylation status and antagonizing the inhibitory phosphorylation function of PKC412 is more associated to the FLT3 positive leukemic cell population. Thus, these novel results suggest that FLT3 surface expression is involved in the intracellular phosphorylation effects of cytarabine.

## Discussion

FLT3-ITD is a common mutation of AML and results in constitutive activation of FLT3. This in turn provides a survival advantage of leukemic cells by activation of multiple downstream effector molecules. FLT3-ITD is associated with worse prognosis and resistance to chemotherapy [5, 26]. In our study, leukemic cells withdrawn from blood of two AML patients with mutated FLT3 who received induction therapy with daunorubicin and cytarabine showed a prominent increase in FLT3 surface expression already at the first three days of treatment (Fig. 4). This is in line with our in vitro data, identifying increased surface localization of FLT3 on MV4-11 and MOLM-13 cells after cytarabine treatment (Figs. 2 and 3). Our study suggests that the level of FLT3 expression is coupled to the intracellular therapy response of cytarabine, which subsequently leads to increased phosphorylation of several vital signaling proteins. As a consequence, cytarabine could potentially antagonize the inhibitory phosphorylation function of PKC412. To note, cytarabine in combination with PKC412 results in successful treatment in a variety of other model systems and remains a mainstay of AML therapy [27–29]. It has, however, been reported that cytarabine in combination with PKC412 showed antagonism in proliferation assays/combination studies in several cell lines expressing FLT3-ITD [16, 30]. Although apoptosis in the short-term cell cultures was not antagonistically affected in cytarabine treated cells and based on in vitro data, it has been shown that *FLT3* is up-regulated in low responders to cytarabine compared to high responders in long-term xenograft transplantations [31].

Our study focused on FLT3-mutated AML cells, but it would be of interest to investigate FLT3 surface expression after cytarabine treatment in FLT3 mutation-negative AML cells. A study by Farge et al. showed that 12 out of 15 cytarabine low responder patient samples was FLT3 mutated while only 3/10 was FLT3 mutated in the high responder compartment [31]. That make us hypothesize that there is a connection between cytarabine chemoresistance and FLT3-mutated positive cells. In combination with the study from Sato et al. [25], reporting that FLT3 ligand in plasma of FLT3-ITD AML patients is highly increased during intensive chemotherapy, this could be a plausible mechanism for FLT3 signaling pathway to be involved in therapy resistance of leukemic cells. When fractionated into two subpopulations of FLT3 high and low expressing cells, we could demonstrate that increased phosphorylation of cytarabine treated cells correlated with the FLT3 high expressing cell population. While this was seen for pAKT, pERK1/2 and pMAPKAPK2 expression, pSTAT5 displayed similar expression level in both FLT3 high and low expressing cells (Fig. 5). This is in agreement with elevated levels of FLT3 surface expression and the fact that previous reports have demonstrated that FLT3-ITD localized intracellularly at the endoplasmic reticulum (ER) or at the cell membrane exhibit qualitative differences in signal transduction. While FLT3-ITD at the ER independently of cytokine stimulation activates STAT5, cell surface localized FLT3-ITD leads to activation of the MAPK/AKT and PI3K/ERK pathways but not STAT5 [32–34]. Moreover, Chen and colleagues [33] showed that FLT3 on the cell surface is indispensable to FL-induced resistance to therapy and reduced surface expression of FLT3 attenuated the FL induced resistance. This suggest that the expression levels of FLT3 on the cell surface is critical for the intracellular responsiveness of therapy. While the data presented here do not provide direct evidence of any relationship between the phosphorylation effects and apoptosis, the results support the idea that FLT3 expression is involved in development of chemotherapy-resistance in AML cells and suggest a possible mechanism of elevated surface expression of FLT3 and increased phosphorylation status of cytarabine treated cells.

The combination of PKC412 and daunorubicin has previously been shown to have synergistic effects [16, 30]. The pharmacokinetic interaction between the two drugs has been suggested as a plausible explanation, but little is known about the underlying mechanisms [9]. Although the exact mechanism of action is still unclear, we show that daunorubicin synergistically/adaptively inhibits protein phosphorylation when administered together with PKC412. Promising on-going clinical trials as well as pre-clinical studies strongly support further investigation of PKC412 in combination with other agents for AML patients to be crucial for development of novel therapies [10, 16, 30, 35]. Downstream signaling pathways are likely to play an important role in the therapy response, thus deeper understanding about these mechanisms could provide new therapeutic targets and result in new treatment strategies for treatment of AML patients.

## Conclusions

In summary, our results demonstrate that daunorubicin and PKC412 work in a synergistic/adaptive manner to inhibit protein phosphorylation. In contrast, cytarabine antagonizes the inhibitory phosphorylation function of PKC412, possibly through a mechanism involving increased surface localization of FLT3. Building on these findings, further investigations of novel treatment strategies of PKC412 in FLT3-ITD positive AML patients is of interest, and blocking of FLT3 ligand-receptor interaction as well as crucial signaling pathways could be important tools to increase the treatment response and reduce the risk of chemotherapy resistance.

## Supplementary Information

**Additional file 1: Table S1.** Key resources table for mass cytometry analysis. (A) Cell surface markers, (B) intracellular markers. **Table S2.** Calculated transformed ratio of medians by first row using panel/channel values (analysis platform Cytobank; www.cytobank.org).

## Data Availability

The datasets used and/or analyzed during the current study are available from the corresponding author on reasonable request, please contact emma.rorby@liu.se.

## Abbreviations

AC220: Quizartinib
AR: Allelic ratio
AML: Acute myeloid leukemia
AraC: Cytarabine
Dauno: Daunorubicin
ER: Endoplasmic reticulum
FLT3: Fms-related tyrosine kinase 3
FLT3-ITD: Internal tandem duplication of FLT3
FL: FLT3 ligand
MAPK: Mitogen-activated protein kinase
PI3K: Phosphatidylinositol 3-kinase
PKC412/PKC: Midostaurin
TKI: Tyrosine kinase inhibitor
