# Supplementary material for: Multiplexed single‐cell mass cytometry reveals distinct inhibitory effects on intracellular phosphoproteins by midostaurin in combination with chemotherapy in AML cells
Source: Exp Hematol Oncol. 2021 Feb 2;10:7. doi: 10.1186/s40164-021-00201-w (PMC7852110; doi:10.1186/s40164-021-00201-w)
Supplement: Supplementary file 1 — Additional file 1: Table S1. Key resources table for mass cytometry analysis. (A) Cell surface markers, (B) intracellular markers. Table S2. Calculated transformed ratio of medians by first row using panel/channel values (analysis platform Cytobank; www.cytobank.org). [file 40164_2021_201_MOESM1_ESM.docx]

**SUPPLEMENTARY INFORMATION**

**Additional file 1**

**Table S1**

**Table S2**
